# Experimental investigation of the effects of acute exercise on memory interference

**DOI:** 10.15171/hpp.2018.28

**Published:** 2018-07-07

**Authors:** Savanna Wingate, Lindsay Crawford, Emily Frith, Paul D. Loprinzi

**Affiliations:** Exercise Psychology Laboratory, Physical Activity Epidemiology Laboratory, Department of Health, Exercise Science and Recreation Management, The University of Mississippi, University, MS 38677, USA

**Keywords:** Acquisition, Consolidation, Encoding, Memory, Physical activity

## Abstract

**Background:** Among other factors, including the decay theory, interfering stimuli (proactive and retroactive interference; PI and RI) may influence the encoding and consolidation of target information. Acute exercise can enhance episodic memory function, but no experiments have evaluated whether exercise can attenuate PI and RI effects on memory, which was the purpose of this experiment.

**Methods:** Twenty young adults were randomized (via computer program) into one of 6 experimental groups (N=120, n=20 per group), including 3 PI (G1, G2, and G3) and 3 RI groups (G4, G5, and G6). Those in G1 and G4 exercised prior to a 10-list AB/AC paradigm with interference; G2 and G5 did not exercise but had interference; and G3 and G6 were the control groups with no exercise and no interference.

**Results:** The mean (95% CI) number of correctly recalled word pairs across the 6 respective groups was 2.4 (1.2-3.5), 2.4 (1.3-3.5), 5.1 (3.9-6.3), 6.9 (5.7-8.0), 5.0 (4.2-5.8), and 6.1 (5.1-6.9) (FANOVA=11.7; P<0.001; η2=0.33). For PI, the control group (group 3) correctly recalled more word pairs (5.1) when compared to the exercise interference group (2.4; group 1) or the non-exercise interference group (2.4; group 2). The difference between group 1 and 3 (2.4 vs.5.1) was significant (P=0.003), as was group 2 vs. 3 (P=0.002). For the RI groups (groups 4-6),group 4 differed from group 5 (6.9 vs. 5.0; P=0.01), but there was no difference between group 4 and group 6 (P=0.25) or group 5 and group 6 (P=0.09).

**Conclusion: **These preliminary findings suggest that acute exercise may be more beneficial for RI compared to PI, but additional experimental work is needed.

## Introduction

The durability of an episodic memory depends on the extent to which the event was encoded as well as factors occurring during the consolidation of the memory. For example, instability of the memory may result from the biological substrate encoding the engram to disintegrate or decay with time; the learned information may be processed in a way that results in erasing of part of the engram; other information may alter the engram or interfere with its expression; critical retrieval cues may fail to be employed; and/or employing inappropriate processing during memory retrieval may influence memory recall.^1^

Among other factors, including the decay theory,^2^ interfering stimuli may influence the encoding and consolidation of target information. Proactive interference occurs when the competing stimuli precedes the target information to be learned, whereas retroactive interference (RI) occurs when the competing stimuli comes after the material to be learned. The standard paradigm for assessing memory interference is the AB/AC paradigm, which suggests that memory is worse for associations (AB trials) when one member of the association is shared in a subsequent trial (AC trials) then for associations where a member is not subsequently represented (DE trials). Mechanisms of this effect may include item suppression, candidate competition and associative competition effects. Emerging work demonstrates that memory reactivation, or the reinstatement of processes and representations engaged when an event is initially experienced, may help increase resistance to RI.^3^ Although speculative, this may occur by providing an opportunity to re-encode and thus strengthen the association between the word and its original encoding task.^3^ Additionally, emerging work demonstrates that the medial prefrontal cortex plays a critical role in minimizing interference effects by helping to discriminate among conflicting representations.^4^

Recent work demonstrates that acute exercise engagement may help to facilitate episodic memory function.^5-10^ Specifically, exercise prior to the memory encoding may help to facilitate CREB-1 levels^11^ as well as neuronal excitability,^12,13^ thus helping to prime neuronal cells into encoding a particular memory.^14^ Additionally, enhanced attention may facilitate memory encoding via modulation of the dopamine neurotransmitter acting on D1/D5 receptors,^15^ with animal work showing that exercise can increase the expression of dopamine and dopamine receptors,^16,17^ possibly through DA-D_2_R protein expression.^18,19^ Additionally, exercise has been shown to upregulate AMPA receptor levels,^20,21^ open NMDA channels,^22^ phosphorylate glutamate receptors, and increase EPSP in the hippocampus,^20^ which are key characteristics of facilitating memory-related long-term potentiation.^23^

What has yet to be investigated is whether acute exercise can minimize a memory interference effect, as assessed via the AB/AC paradigm. Such an effect is plausible given the observed effect that acute exercise has on post-exercise neuronal activity in the prefrontal cortex,^24^ coupled with the effect the prefrontal cortex plays in minimizing a memory interference effect.^4^ Thus, the purpose of this study was to examine whether acute moderate-intensity exercise can reduce proactive- and retroactive-memory interference. We hypothesize that, compared to a non-exercise memory interference group, a short bout of acute moderate-intensity exercise will reduce proactive- and retroactive-memory interference, and thus, enhance short-term associative memory. This may have important health promotion implications as memory function is vital for optimal daily functioning.^25^

## Materials and Methods

### Study design

All data collection occurred between August and December of 2017, with all data collection occurring in the authors’ Exercise Psychology Laboratory. The present study is a 6-arm, parallel, between-group randomized controlled trial. Randomization was employed using a computer-generated program. See Table 1 for an overview of the study design and for how proactive and retroactive interference was assessed. For proactive interference, three groups were assessed (groups 1-3), including 2 experimental arms and a control arm. Experimental arm 1 involved exercising prior to the AB/AC paradigm; experimental arm 2 involved no exercise prior to the AB/AC paradigm; and the control arm involved no interference effect, i.e., learning AC and then recalling AC. Groups 4-6 were identical to groups 1-3 except RI was assessed (Table 1).

The exercising groups (groups 1 and 4; Table 1) consisted of engaging in an acute 15-minute bout of moderate-intensity exercise, resting for 5 minutes in a seated position, and then completed the AB/AC paradigm. The remaining groups sat quietly for 20 minutes and then completed the AB/AC paradigm. Additional details to follow.

### Participants 

Each group included approximately 20 participants (college students), which aligns with our other related experimental work.^6-10^ With 6 groups total, the total sample size included 120 participants. Participants were recruited through classroom announcements and word-of-mouth. Participants included male and females from the ages of 18 to 35 years. Additionally, participants were excluded if they self-reported as a daily smoker,^26,27^ self-reported being pregnant,^28^ exercised within 5 hours of testing,^5^ consumed caffeine within 3 hours of testing,^29^ had a concussion or head trauma within the past 30 days,^30^ took marijuana or other illegal drugs within the past 30 days,^31^ were considered a “heavy” alcohol user (>30 drinks/month for women; >60 drinks/month for men),^32^ or were left-hand dominant or mixed-handed.^33^

### Exercise protocol

Those randomized to the exercise group walked on a treadmill for 15 minutes at a self-selected “brisk walking” pace. Specifically, they were told to “self-select a brisk walking pace, a pace as if you were late for class; please ensure that the speed is at least 3.0 mph and maintain this speed throughout the treadmill walk.” The researcher confirmed that the speed was maintained throughout the exercise bout. This exercise has previously been shown to enhance episodic memory.^34^

### Memory assessment

Procedures of this employed AB/AC paradigm are described elsewhere.^35^ Randomly selected word pairs from the Toronto Word Pool were used. In the initial study trial (List 1), 10 pairs were presented one at a time on a computer screen for 5-seconds each. Three cycles of this occurred. Immediately after viewing List 1 for the third cycle, participants began the second list, using the same procedure as described for List 1 (see Table 2 for word pairs that were used for List 1 and List 2). After studying List 2, participants performed a distractor task for 5 minutes. This involved watching a 5-minute video clip of the TV show “The Office” and then rating the humor level of this clip.

Immediately after this distractor task, participants performed a cued-recall assessment. As noted in Table 1, groups 1-3 were asked to perform a cued-recall of List 2, whereas groups 4-6 were asked to perform a cued-recall of List 1. The cued-recall involved one of the first words from a randomly selected pair from the appropriate list being presented on a computer screen for 20 seconds. The participant then wrote down the second word from that pair in a booklet. This continued until all 10 pairs were completed.

The outcome of interest was the number of correctly recalled pairs (max score is 10). For the proactive interference procedures (Table 1), we anticipated that the control scenario (group 3) would have the highest score. We then anticipated that, among the 2 proactive interference experimental arms (groups 1 and 2), those who exercised prior to List 1 (group 1) would have a reduced proactive interference effect when compared to group 2, and thus, group 1 would correctly recall more pairs than group 2. We anticipated the same trend for retroactive interference.

### Physical activity assessment

Habitual self-reported moderate-to-vigorous physical activity (MVPA) assessment^36^ was measured using the Physical Activity Vital Sign instrument. Participants self-reported the number of minutes per week engaged in MVPA. This assessment has demonstrated evidence of validity.^37-41^ Notably, this self-report MVPA measure correlates with accelerometer-assessed number of days > 30 bout-min MVPA (*r* = 0.52, *P* < 0.001).^38^

### Mood assessment

A mood state survey (PANAS) was used to assess overall mood state.^42^ Regarding the Positive and Negative Affect Schedule (PANAS),^42^ participants rated 20 items (e.g., excited, upset, irritable, attentive) on a Likert scale (1, very slightly or not at all; to 5, extremely), with half of the items constituting a “positive” mood state, with the other half being a “negative” mood state. In this sample, for the positive and negative mood states, respectively, Cronbach’s alpha was 0.90 and 0.82. For potential confounding purposes, we assessed these variables (physical activity and mood) to determine the similarities of these parameters across the 6 experimental groups.

### Statistical analysis

All analyses were computed in SPSS (v. 22, IBM SPSS, Armonk, NY, USA). An analysis of variance (ANOVA) test was computed to examine cued-recall scores across the groups, with Bonferroni post hoc tests employed. All assumptions (independence of cases, normality and equality of variances) of this analytical test were assessed and determined not to be violated. Statistical significance was established as a nominal alpha of 0.05.

## Results

Characteristics of the sample, across the 6 experimental groups, are shown in Table 3. Participants were similar across the demographic, behavioral, physiological (resting heart rate), and psychological assessments (mood state). The 2 exercise groups had a similar exercise-induced physiological response (exercise heart rate).

**Figure 1:**
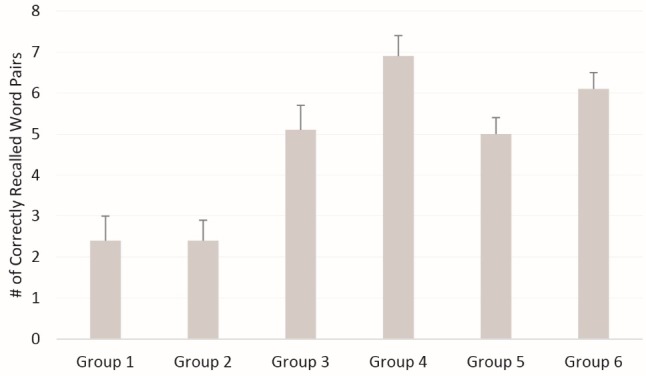
Number of correctly recalled word pairs across the 6 experimental groups.

Figure 1 displays the mean number of correctly recalled word pairs across the 6 experimental groups (numeric values also shown in Table 3). The mean (95% CI) number of correctly recalled word pairs across the 6 respective groups was 2.4 (1.2-3.5), 2.4 (1.3-3.5), 5.1 (3.9-6.3), 6.9 (5.7-8.0), 5.0 (4.2-5.8), and 6.1 (5.1-6.9) (F_ANOVA_=11.7; *P*< 0.001; η^2^= 0.33). As expected, for proactive interference the control group (group 3) correctly recalled more word pairs (5.1) when compared to the exercise interference group (2.4; group 1) or the non-exercise interference group (2.4; group 2). The difference between Group 1 and 3 (2.4 vs. 5.1) was statistically significant (*P*= 0.003), as was group 2 vs. 3 (*P*= 0.002). For the retroactive interference groups (groups 4-6), group 4 statistically differed from group 5 (6.9 vs. 5.0; *P*= 0.01), but there was no statistical difference between group 4 and group 6 (*P*= 0.25) or group 5 and group 6 (*P*= 0.09).

## Discussion

The purpose of this experiment was to examine the effects of acute exercise on episodic memory function while manipulating a potential interference effect. The motivation for this experiment was 2-fold: (1) interfering stimuli (PI or RI) can influence memory retrieval, and (2) acute exercise has been shown to improve episodic memory function without competing stimuli. In this experiment, we were able to induce a proactive interference effect, as demonstrated by group 3 correctly recalling more word pairs than groups 1 and 2. Acute exercise did not minimize a proactive interference effect, as groups 1 and 2 correctly recalled a similar number of words (2.4). Regarding retroactive interference, group 6 (6.1) correctly recalled more words than group 5 (5.0), suggesting that a retroactive interference effect was observed. However, this difference was not statistically significant, suggesting that this retroactive interference effect was not completely observed. Relying on statistical significance, however, is a questionable practice.^43-45^ Interestingly, group 4 correctly recalled more words than group 5 (6.9 vs. 5.0), providing some suggestive evidence that acute exercise may help to minimize a retroactive interference effect. Until future confirmatory work is completed, such a possibility, however, should be interpreted cautiously.

Although not in the context of a cognitive-related episodic memory task, recently, Lauber et al^46^ evaluated whether a single session of high-intensity interval training (HIIT) can mitigate the effects of an interfering motor task. Participants performed ballistic training and then the HIIT either before or after practicing an interfering accuracy motor task. After ballistic training, all groups increased their performance in the trained and untrained limb. However, despite practicing the interfering task, the HIIT bout before the interfering task maintained their ballistic performance, whereas the group that engaged in HIIT after the interfering task showed an interference effect. This suggests that exercise prior to an interference task may help to minimize a motor memory interference effect. The findings of our experiment did not demonstrate evidence that acute moderate-intensity exercise can reduce a proactive interference effect, but our findings provide some support that acute exercise may be useful in mitigating a retroactive interference effect. Compared to those who did not exercise (group 5), those who exercised (group 4) before the training list (List 1) had a greater cued-recall on List 1 even after being exposed to List 2 prior to the recall. This observation aligns with other work demonstrating that acute exercise can improve working memory performance,^47-51^ which has a similar temporal protocol (i.e., exposed to a target item, then exposed to a competing stimulus, and then recall the target item) to that of our retroactive interference protocol.

Acute exercise may help to minimize a retroactive interference effect by enhancing markers of working memory (e.g., BDNF, post-synaptic density protein 95^52^). Catecholamines, such as dopamine and norepinephrine play a critical role in working memory capacity.^53^ For example, D1 receptor stimulation enhances the excitability of prefrontal pyramidal cells and potentiates glutamate gated currents.^53^ Importantly, working memory is optimized at intermediate levels of D1 receptor stimulation and is degraded by either too little, or too much activation of this dopamine receptor.^53^ Given the exercise-induced effects on dopamine production,^17^ moderate-intensity exercise, in particular, may help to optimize changes in these molecular mediators of working memory capacity. Additionally, psychological models have been developed as a potential mechanistic explanation for individual differences in working memory capacity. Relatedly, cognitive attention plays a critical role in working memory capacity,^53^ and in theory, moderate-intensity exercise intensity may have an optimal effect on attention-influenced working memory capacity.^9^

In conclusion, our findings provide some suggestive evidence that acute exercise may, partially, minimize a retroactive interference effect. Future confirmatory work is needed, and if confirmed, mechanistic work will be needed to identify why exercise may help to minimize retroactive interference but not influence proactive interference. Despite the strengths of this study, which includes its novelty, comprehensiveness, and experimental design approach, future work should overcome the limitations of this study. A limitation includes the relatively small sample size per group. Additionally, future work should consider a within-subject design. The major source of outcome variance in between-subject designs is individual differences among the participants, and a within-subject design may help to minimize this outcome heterogeneity and potentially maximize exercise-induced effects. Such a within-subject design should consider other techniques to evaluate proactive and retroactive interference, such as using Yule’s Q for associative interference.^54,55^ If such future replicative work provides robust evidence that exercise can minimize a memory interference effect, then this may have important health promotion implications, as memory function is vital for optimal daily functioning.^25^

## Ethical approval

All procedures performed in studies involving human participants were in accordance with the ethical standards of the institutional and/or national research committee and with the 1964 Helsinki declaration and its later amendments or comparable ethical standards. This study was approved by the University of Mississippi institutional review board (#18-001), with informed consent obtained from all individual participants included in the study.

## Competing interests

The authors declare that they have no competing interests.

## Authors’ contributions

SW and LC collected the data. PL performed analyses and drafted the manuscript. All authors have reviewed and provided intellectual feedback on the manuscript. All authors have read and approved the final version of the manuscript and agree with the order of presentation of the authors.

**Table 1:** Study design to assess proactive and retroactive interference

**Group No.**	**Group Classification**	**Proactive Interference**		
		**Learn List 1**	**Learn List 2**	**Cued-Recall List 2**
1	Experimental 1 – Exercise	A-B	A-C	A-C
2	Experimental 2 – No Exercise	A-B	A-C	A-C
3	Control – No Exercise		A-C	A-C
		**Retroactive Interference**		
		**Learn List 1**	**Learn List 2**	**Cued-Recall List 1**
4	Experimental 1 – Exercise	A-B	A-C	A-B
5	Experimental 2 – No Exercise	A-B	A-C	A-B
6	Control – No Exercise	A-B		A-B

Six independent groups for this experiment (N = 120). Experimental arm 1 involves exercising prior to learning List 1; Experimental arm 2 involves no exercise prior to learning List 1; Control arm involves no interference effect. The same design occurs for experimental arms 4-6.

**Table 2:** Paired word lists

**List 1: A-B List**		**List 2: A-C List**	
**A**	**B**	**A**	**C**
Belief	Mother	Belief	Legend
Ticket	Hero	Ticket	Duty
Penny	Pursuit	Penny	Patience
Endure	Wrinkle	Endure	Liquid
Wander	Against	Wander	Pupil
Sincere	Funny	Sincere	Belong
Invent	Survive	Invent	Former
Rapid	Column	Rapid	Discuss
Series	Pillow	Series	Because
Fever	Hammer	Fever	Noble

**Table 3:** Characteristics of the sample across the 6 groups

**Variable**	**Group 1**	**Group 2**	**Group 3**	**Group 4**	**Group 5**	**Group 6**
Number	20	21	20	19	20	20
Age (y), mean	19.9 (0.3)	21.0 (0.3)	20.7 (0.3)	20.7 (0.6)	20.8 (0.2)	20.4 (0.3)
Men, %	10.0	23.8	30.0	15.8	30.0	40.0
White, %	70.0	66.6	80.0	73.6	75.0	55.0
Waist circumference, mean cm	83.5 (2.7)	82.2 (2.1)	81.9 (3.0)	78.6 (4.0)	85.0 (3.4)	85.3 (2.3)
MVPA, mean min/wk	241.5 (38.4)	187.8 (35.4)	199.2 (35.8)	250.0 (48.7)	148.7 (27.4)	244.0 (48.7)
Mood, mean positive	30.2 (2.1)	27.2 (1.7)	30.6 (1.7)	29.9 (2.3)	29.6 (2.0)	30.2 (1.9)
Mood, mean negative	12.1 (0.7)	12.6 (0.8)	11.7 (0.4)	13.3 (0.9)	15.9 (1.3)	14.3 (1.0)
Resting HR, bpm	82.3 (2.2)	77.0 (3.2)	76.5 (2.5)	76.6 (3.7)	77.9 (2.7)	79.7 (2.4)
End of Exercise HR, bpm	115.9 (5.7)	-	-	112.8 (5.8)	-	-
Memory Recall, mean No. of correctly recalled word pairs	2.4 (0.6)	2.4 (0.5)	5.1 (0.6)	6.9 (0.5)	5.0 (0.4)	6.1 (0.4)

Abbreviations: MVPA, moderate-to-vigorous physical activity, HR, heart rate; BPM, beats per minute. Participants randomized into one of 6 experimental groups (N=120), including 3 Prospective Interference (G1, G2, and G3) and 3 Retrospective Interference groups (G4, G5, and G6). Those in G1 and G4 exercised prior to the AB/AC paradigm with interference; G2 and G5 did not exercise but had interference; and G3 and G6 were the control groups with no exercise and no interference. Variance estimates in parentheses are standard error values.
